# Establishment and validation of a clinical prediction model for in-stent restenosis after intracranial and extracranial stent implantation

**DOI:** 10.3389/fneur.2025.1516274

**Published:** 2025-05-09

**Authors:** Xiaohan Liang, Kuochang Yin, Yidian Fu, Guodong Xu, Xiaoxiao Feng, Peiyuan Lv

**Affiliations:** ^1^Graduate School of Hebei North University, Zhangjiakou, Hebei, China; ^2^Department of Neurology, Hebei General Hospital, Shijiazhuang, Hebei, China; ^3^Graduate School of Hebei Medical University, Shijiazhuang, Hebei, China; ^4^Hebei Provincial Key Laboratory of Cerebral Networks and Cognitive Disorders, Shijiazhuang, China; ^5^Department of Neurology, Shijiazhuang People’s Hospital, Shijiazhuang, Hebei, China

**Keywords:** restenosis, nomogram, clinical prediction model, cerebral artery stent implantation, TyG

## Abstract

**Objective:**

This study aims to analyze the risk factors for in-stent restenosis in patients who have undergone successful cerebral artery stent implantation and to develop a nomogram-based predictive model.

**Methods:**

We utilized data retrospectively collected from 488 patients at Hebei Provincial People’s Hospital between April 2019 and March 2024. After applying the inclusion criteria, 390 patients were further analyzed and divided into a training group (*n* = 274) and a validation group (*n* = 116). In the training group, we used univariate and multivariate logistic regression to identify independent risk factors for stroke recurrence and then created a nomogram. The nomogram’s discrimination and calibration were assessed by examining various metrics, including the concordance index (C-index), the area under the Receiver Operating Characteristic (ROC) curve (AUC), and calibration plots. Decision curve analysis (DCA) was employed to evaluate the clinical utility of the nomogram by quantifying the net benefit for patients at different probability thresholds.

**Results:**

The nomogram for predicting in-stent restenosis in patients undergoing cerebral artery stenting included seven variables: triglyceride-glucose index (TyG), presence of Diabetes Mellitus, postoperative dual antiplatelet therapy, body mass index (BMI), and preoperative MRS score. The C-index (0.807 for the training cohort and 0.804 for the validation cohort) indicated satisfactory discriminative ability of the nomogram. Furthermore, DCA indicated a clinical net benefit from the nomogram.

**Conclusion:**

The predictive model constructed includes six predictive factors: TyG, presence of Diabetes Mellitus, postoperative dual antiplatelet therapy, BMI, and preoperative MRS score. The model demonstrates good predictive ability and can be utilized to predict ischemic stroke recurrence in patients with symptomatic ICAS after successful stent placement.

## Introduction

A report from the American Heart Association indicates that cerebral ischemia is one of the leading causes of death in industrialized countries, also contributing to numerous medical and economic challenges (1). Cerebral vascular stenosis or occlusion is a major cause of cerebral ischemia. With advancements in neurointerventional techniques, stent-assisted angioplasty has emerged as a viable option for select cases of intracranial/extracranial stenosis refractory to medical therapy (2, 3). In-stent restenosis (ISR) results from three primary pathological mechanisms: neointimal hyperplasia, atherosclerotic plaque progression, and thrombus formation (4). Studies have shown that in-stent restenosis is determined by either excessive tissue hyperplasia within the stent lumen or a *de novo* atherosclerotic process (5). Stent implantation and endothelial injury can lead to atherosclerotic plaque rupture, where the interaction of platelets, fibrin, and other components triggers an inflammatory response. This process involves mediators such as platelet-derived growth factor (PDGF), basic fibroblast growth factor, and transforming growth factor-beta (TGF-β), which promote neointimal hyperplasia and contribute to in-stent restenosis (6). Early in-stent neointimal hyperplasia and later atherosclerosis significantly affect the longevity of the stent, greatly impacting the long-term prognosis of patients (7, 8). Neointimal hyperplasia and atherosclerosis can lead to restenosis or occlusion within the stent, a condition known as in-stent restenosis (ISR). This may result in more severe ischemic diseases, making the occurrence of ISR a crucial prognostic indicator for stent implantation. One study demonstrated that patients with Intracranial Stent Restenosis exhibited higher long-term stroke incidence and mortality rates, with ISR being associated with an increased proportion of clinical complications (9).

Reports indicate that the incidence of restenosis following carotid artery stenting ranges from 5 to 20% (10). Restenosis after stent implantation is caused by multiple factors. A multicenter study suggests that in-stent restenosis is associated with factors such as age, gender, history of prior neck irradiation, prior peripheral bypass surgery, and whether post-dilation was performed after stent placement (11). Diabetes has also been shown to be a significant risk factor for in-stent restenosis (12). In recent years, several blood biomarkers have been found to have a significant correlation with in-stent restenosis. Several studies have found that elevated TyG, high-sensitivity C-reactive protein (hs-CRP), and plasma atherogenic index are associated with the occurrence of ISR (8, 13–15). Additionally, the inflammatory response is also one of the causes of ISR (7, 16). Smoking can promote inflammation and increase brain damage by increasing the oxidative stress response of mitochondria (17). Some studies have also suggested that postoperative residual stenosis and collateral circulation compensation may be associated with the occurrence of ISR (18, 19).

Currently, there is growing attention on stroke risk prediction. For example, the recently developed SAMMPRIS-ISR model demonstrate high specificity in forecasting ISR incidence (20). At present, there is limited research on predicting restenosis following cerebral artery stenting. Therefore, this study aims to analyze the factors associated with restenosis in patients undergoing stent implantation, identify potential risk factors, and establish and validate a new predictive model.

## Methods

### Patients selection

We retrospectively collected data from 488 patients who underwent intracranial or extracranial stent implantation at Hebei Provincial People’s Hospital between April 1, 2019, and March 31, 2024.

The inclusion criteria were as follows: (1) The patients were over 18 years of age; (2) Asymptomatic patients with cerebral vascular stenosis ≥70% or symptomatic patients with cerebral vascular stenosis ≥50%, the degree of stenosis was measured by Digital Subtraction Angiography (DSA) of cerebral vascular, based on the North American Symptomatic Carotid Endarterectomy Trial (NASCET) criteria; (3) The patient or their legal guardian provided informed consent and signed the specific consent form for cerebral stent implantation; (4) Inclusion was restricted to patients with clinical indications for solitary stent placement; and (5) A follow-up DSA of the cerebral vessels was performed 6 months later.

The exclusion criteria were as follows: (1) Continued use of anticoagulants for heart disease; (2) Discontinuation of antiplatelets for more than 2 weeks due to bleeding tendency such as in patients with a chronic subdural hematoma or a gastric ulcer; (3) Patients who did not undergo follow-up DSA of the cerebral vessels after 6 months; (4) Patients who experienced adverse events during the surgery (vascular dissection at the surgical site, distal occlusion, or intracranial hemorrhage during the procedure); and (5) Patients with Cancer, heart failure, renal failure, and other similar severe systemic conditions.

All images were reviewed by two interventional neuroradiologists, and any discrepancies were confirmed by a third doctor. This prospective observational study was approved by the Ethical Committee of Hebei General Hospital (2024-LW-0180).

Medical treatment. Due to the retrospective nature of this study, which utilized patient medical record data, written informed consent was not obtained from the participants.

All patients received dual antiplatelet therapy within 24 h after the stent procedure (aspirin 100 mg/day and clopidogrel 75 mg/day, or aspirin 100 mg/day and ticagrelor 90 mg twice daily). All patients underwent platelet function testing using platelet aggregation rate assessments. A platelet aggregation rate of less than 42.9% was considered indicative of effective dual antiplatelet therapy (21). This means the patient meets the criteria for stent surgery. All patients were required to continue dual antiplatelet therapy for at least 3 months after the surgery, followed by lifelong aspirin (100 mg/day) therapy. Postoperatively, patients routinely used statins to control lipid levels, with the choice of medication based on the patient’s lipid metabolism levels.

### Procedures

All extracranial stent procedures were performed with the patient awake, while intracranial interventions were performed under general anesthesia. Intraoperative anticoagulation was achieved using 100 units/kg heparin, all patients were fully anticoagulated with heparin before the placement of the guiding catheter. The procedure was performed with the patient in the supine position, using the femoral artery for cerebral artery stenting. First, a balloon was advanced through the stenosis along a microcatheter. An appropriately sized balloon was selected and inflated at the stenosis site, left in place for 0.5 min, if post-dilatation imaging shows a residual stenosis greater than 50%, a second dilatation may be performed. A stent of suitable diameter was chosen based on the vessel size, and a stent covering the length of the stenosis was selected. The stent was slowly advanced through the stenosis and deployed at the appropriate location. Angiography showed no significant restenosis at the stent site, and a closure device was used to achieve hemostasis at the femoral artery. The definition of a successful angioplasty is when the degree of stenosis is less than 30% after stent placement (12). The Neuroform EZ (Stryker) or Enterprise VRD (Codman Neuro) was selected for intracranial stenting, whereas extracranial segments were treated with Wallstent (Boston Scientific) or Protégé (ev3) stent systems.

### Data collection and definition

Patient characteristics that were recorded included demographic data, risk factors for cerebral infarction, and clinical data. The primary endpoint was ISR. The endpoint was determined by an experienced interventional neuroradiologist after a comprehensive review of the cerebral DSA. In-stent restenosis was defined as a stenosis degree of 50% or greater within the stent or in the 5 mm margins adjacent to the stent (14). The collected data included age, sex, BMI, blood pressure, current smoking status (yes or no), alcohol consumption (yes or no), medical history (hypertension, dyslipidemia, diabetes mellitus, ischemic stroke and coronary heart disease), fasting laboratory data [total cholesterol, TG, high-density lipoprotein cholesterol (HDL-C), low-density lipoprotein cholesterol (LDL-C), FBG, platelet, neutrophil, lymphocyte, uric acid, homocysteine, white blood cells, the diameter and length of the stent, stent deployment site (intracranial or extracranial), ADP, anterior and posterior circulations, preoperative the National Institutes of Health Stroke Scale score, discharge the National Institutes of Health Stroke Scale score, preoperative Modified Rankin Scale score and discharge Modified Rankin Scale score].

The formula for calculating the TyG index was Ln [fasting triglyceride (mg/dL) × fasting blood glucose (mg/dL)/2] (14). The AIP is defined as the base 10 logarithm of the ratio of the concentration of TG to HDL-C (8). BMI was calculated as weight/height2 (kg/m^2^). Hypertension was defined as a blood pressure ≥ 140/90 mmHg or reception of antihypertensive therapy. Diabetes mellitus was defined as an FBG > 7.0 mmoL/L, random blood glucose > 11.1 mmoL/L, or reception of hypoglycemic drugs (12). Dyslipidemia was defined as fasting total cholesterol > 6.22 mmoL/L, TG ≥ 2.26 mmoL/L, LDL-C ≥ 4.14, HDL < 1.04 mmoL/L, or reception of lipid-lowering drugs (22). Smoking is defined as current daily smoking or having quit for less than 5 years; non-smoking is defined as never having smoked daily or having quit for more than 5 years. The drinking habit is defined as an average alcohol consumption of ≥2 units/day (16 g/day) for at least the past 6 months, TL refers to the use of ticagrelor or clopidogrel as part of dual antiplatelet therapy (DAPT) after surgery.

### Evaluation of ISR

Evaluate ISR based on follow-up DSA results. The NASCET method was applied to determine the degree of restenosis on DSA images. All images were assessed by experienced interventional neuroradiologists.

### Statistical analysis

Categorical data were expressed as absolute and percentage values. Continuous data with a normal distribution were described using mean ± SD (standard deviation), while non-normally distributed continuous data were expressed as the median and interquartile range (IQR). Multiple imputation was used to fill in missing values. The dataset was randomly divided into a training set and a validation set in a 7:3 ratio. Risk factors with statistical significance (*p* < 0.05) in univariate logistic analysis were considered potential risks and included in multivariate logistic analysis. A predictive model was developed using multivariable logistic regression and visualized with a nomogram. Model selection was optimized through stepwise forward and backward selection based on the Akaike Information Criterion (AIC). The performance of the nomogram was assessed using the concordance index (C-index) and the Receiver Operating Characteristic (ROC) curve, with the Area Under the Curve (AUC) representing the results. Calibration curves were used to compare actual outcomes with predicted probabilities, and decision curve analysis (DCA) evaluated the clinical utility of the model. Data processing was performed using RStudio.

## Results

### Patient characteristics

**Figure 1:**
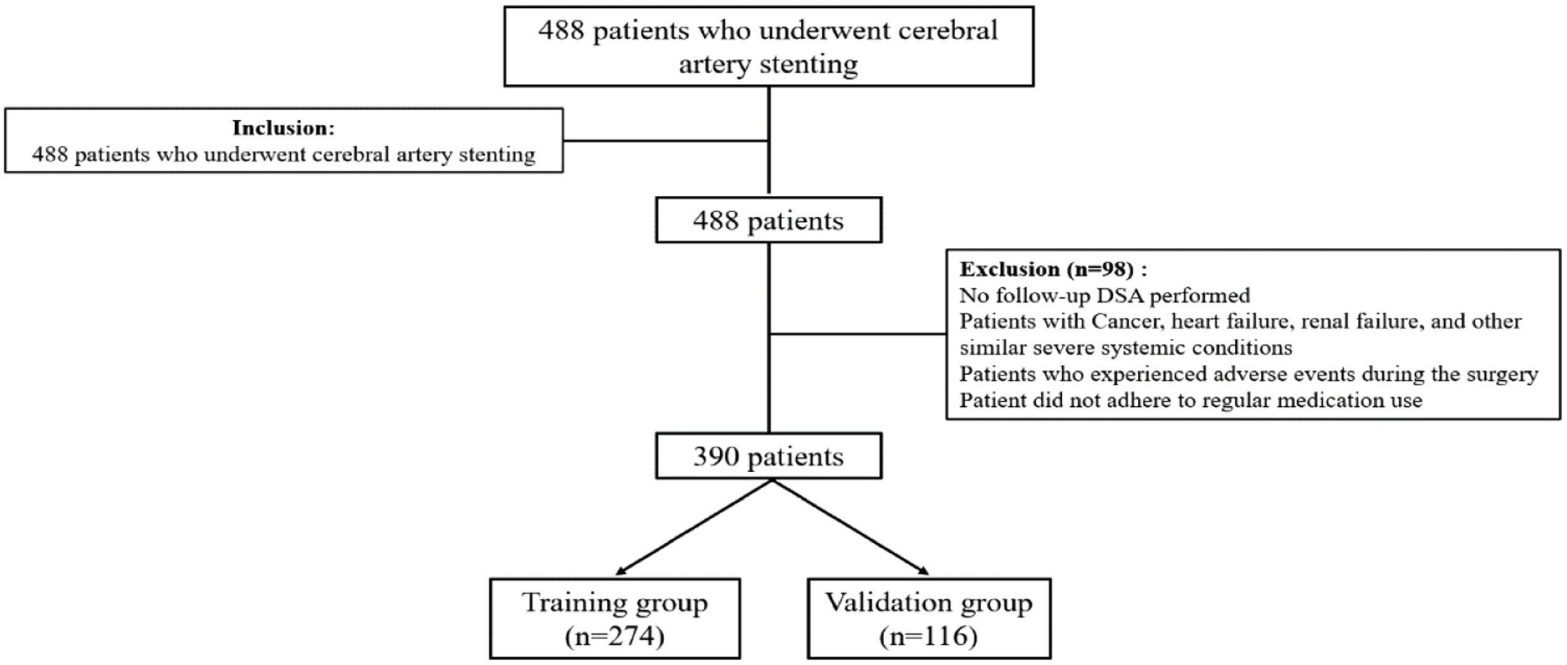
Flowchart of study population.

A total of 488 consecutive patients who underwent stent implantation at our institution from April 2019, and March 2024 were screened, and 390 cases were ultimately eligible for our study. They were further divided into training (*n* = 274) and validation (*n* = 116) groups (Figure 1). The flow diagram of model development and validation is presented in Figure 1. Baseline demographic and clinical characteristics for both the training and validation cohorts are presented in Table 1. The incidence of restenosis in the training group 6 months after stent implantation was 20.1%. The incidence of restenosis in the validation group 6 months after stent implantation was 28.4%.

**Table 1:** Baseline characteristics of participants.

Variables	Overall (*n* = 390)	Training group (*n* = 274)	Validation group (*n* = 116)	*p*
Sex (%)				
Male	239 (61.3)	169 (61.7)	70 (60.3)	0.974
Female	151 (38.7)	105 (38.3)	46 (39.7)	
Age (median [IQR])	58.00 [52.00, 66.00]	58.00 [51.00, 66.00]	59.00 [52.75, 65.00]	0.47
Smoke (%)				
No	305 (78.2)	219 (79.9)	86 (74.1)	0.96
Yes	85 (21.8)	55 (20.1)	30 (25.9)	
Drink (%)				
No	353 (90.5)	255 (93.0)	98 (84.5)	0.981
Yes	37 (9.5)	19 (7.0)	18 (15.5)	
Hypertension (%)				
No	130 (33.3)	87 (32.5)	41 (35.3)	0.567
Yes	260 (66.7)	185 (67.5)	75 (64.7)	
Diabetes_mellitus (%)				
No	291 (74.6)	208 (75.9)	83 (71.6)	0.662
Yes	99 (25.4)	66 (24.1)	33 (29.4)	
CAD (%)				
No	344 (88.2)	246 (89.8)	98 (84.5)	0.669
Yes	46 (11.8)	28 (10.2)	18 (15.5)	
Hyperlipidaemia (%)				
No	375 (96.2)	261 (95.3)	114 (98.3)	0.767
Yes	15 (3.8)	13 (4.7)	2 (1.7)	
Cycle (%)				
Anterior circulation	248 (63.8)	176 (64.2)	72 (61.2)	0.375
Posterior circulation	140 (36.2)	98 (35.1)	44 (38.8)	
Antiplatelet therapy (%)				
Clopidogrel	126 (32.3)	96 (35.0)	30 (25.9)	
Ticagrelor	264 (67.7)	178 (65.0)	86 (74.1)	
plt (median [IQR])	232.50 [194.50, 272.75]	234.50 [194.00, 275.00]	226.00 [197.50, 267.00]	0.641
Neut (median [IQR])	4.22 [3.38, 5.93]	4.22 [3.33, 5.92]	4.27 [3.55, 6.19]	0.374
lymph (median [IQR])	1.64 [1.31, 2.02]	1.61 [1.30, 2.01]	1.68 [1.33, 2.02]	0.315
NLR (median [IQR])	2.52 [1.88, 3.73]	2.55 [1.87, 3.65]	2.50 [1.94, 3.85]	0.913
PLR (median [IQR])	137.37 [107.80, 188.28]	141.27 [110.67, 188.59]	129.47 [105.22, 188.46]	0.333
TC (median [IQR])	4.02 [3.40, 4.88]	3.99 [3.38, 4.83]	4.12 [3.51, 4.96]	0.141
TG (median [IQR])	1.28 [0.96, 1.70]	109.38 [81.48, 149.24]	123.11 [91.89, 154.11]	0.081
TyG (median [IQR])	1.29 [0.92, 1.59]	5.75 [5.36, 6.02]	5.87 [5.50, 6.24]	0.018

Variables	Overall (*n* = 390)	Training group (*n* = 274)	Validation group (*n* = 116)	*p*
HDL (median [IQR])	1.07 [0.91, 1.25]	1.06 [0.91, 1.26]	1.12 [0.95, 1.23]	0.279
AIP (median [IQR])	0.09 [−0.06, 0.22]	0.08 [−0.08, 0.23]	0.10 [−0.03, 0.21]	0.432
LDL (median [IQR])	2.55 [2.02, 3.08]	2.49 [1.98, 3.04]	2.64 [2.20, 3.18]	0.164
UA (median [IQR])	284.50 [228.20, 359.80]	283.95 [228.00, 358.10]	289.70 [233.98, 361.10]	0.744
Hcy (median [IQR])	12.70 [10.20, 16.90]	12.80 [10.10, 16.62]	12.70 [10.38, 17.60]	0.987
WBC (median [IQR])	6.69 [5.32, 8.24]	6.74 [5.20, 8.23]	6.72 [5.81, 8.35]	0.218
FBG (median [IQR])	5.47 [4.72, 6.49]	5.34 [4.70, 6.36]	5.62 [4.82, 7.30]	0.056
lenth (median [IQR])	15.00 [15.00, 20.00]	15.00 [15.00, 20.00]	15.00 [15.00, 20.00]	0.482
diameter (median [IQR])	4.00 [3.50, 4.50]	4.00 [3.50, 4.50]	4.00 [3.50, 4.50]	0.643
Stent site				
Intracranial	309 (79.2)	220 (80.3)	89 (84.0)	0.427
Extracranial	81 (20.8)	54 (19.7)	27 (16.0)	
ADP (median [IQR])	26.95 [17.98, 35.00]	25.65 [17.98, 35.00]	28.35 [17.45, 35.70]	0.469
BMI (mean (SD))	26.20 ± 3.62	26.19 ± 3.64	26.23 ± 3.61	0.865
Innihss (median [IQR])	0.50 [0.00, 3.00]	0.00 [0.00, 1.00]	0.00 [0.00, 1.00]	0.989
Outnihss (median [IQR])	0.00 [0.00, 1.00]	1.00 [1.00, 2.00]	1.00 [0.00, 2.00]	0.489
InMRS (median [IQR])	1.00 [0.25, 2.00]	0.00 [0.00, 1.00]	0.00 [0.00, 1.00]	0.636
OutMRS (median [IQR])	0.00 [0.00, 1.00]	0.00 [0.00, 1.00]	0.00 [0.00, 1.00]	0.695
ISR (%)				
No	302 (77.4)	219 (79.9)	83 (71.6)	0.138
Yes	88 (23.6)	55 (20.1)	33 (28.4)	

SD, standard deviation; IQR, interquartile range; NIHSS, National Institutes of Health stroke scale; MRS, modified Rankin scale; ISR, in-stent restenosis; TG, triglyceride; TC, total cholesterol; LDL, low-density lipoproteins; HDL, high-density lipoproteins; HCY, homocysteine; WBC, white blood cells; ADP, Adenosine Diphosphate; FBG, fasting blood glucose; UA, uric acid; TyG, triglyceride-glucose index; AIP, atherogenic Index of Plasma, antiplatelet therapy ticagrelor or clopidogrel.

### Univariate analysis and multivariate analysis

Univariate logistic regression analysis of the survival of 274 patients in the modeling group is revealed in Table 2. In the univariate logistic regression analysis, 14 variables (PLR, TG, TyG, HDL, AIP, FBG, Hypertension, Diabetes mellitus, the postoperative dual antiplatelet therapy regimen, BMI, Innihss, Outnihss, InMRS, OutMRS all displayed high statistical differences) (Table 2). Due to collinearity between TG, TyG and FBG, therefore, only TyG was retained in the final model, while TG and FBG were excluded due to collinearity (Table 3). Furthermore, candidates for stepwise multivariate logistic regression analyses were selected from variables with a *p* value of less than 0.05 in the univariate logistic analyses. Using the AIC in a multivariable logistic regression model for forward and backward stepwise selection, the following seven variables were identified as most closely associated with recurrence risk: TyG (OR = 4.63, 95% confidence interval (CI): 2.17–9.84, *p* < 0.001), Diabetes mellitus (OR = 4.34, 95% confidence interval (CI): 2.11–8.95, *p* < 0.001), DAPT regimen (OR = 0.44, 95% confidence interval (CI): 0.21–0.93, *p* = 0.031), BMI (OR = 1.13, 95% confidence interval (CI): 1.01–1.26, *p* = 0.026), InMRS (OR = 1.32, 95% confidence interval (CI): 1.01–1.72, *p* = 0.042) (Table 4).

**Table 2:** Univariate logistic regression analysis for stroke recurrence in the training cohorts.

Variables	OR	95 %CI	*p*-value
Sex	0.99	0.54–1.82	0.981
Age	1.00	0.98–1.03	0.808
Smoke	0.86	0.4–1.84	0.696
Drink	0.21	0.03–1.58	0.129
plt	1.00	0.99–1.01	0.874
neut	1.00	0.95–1.06	0.895
lymph	1.08	0.9–1.3	0.410
NLR	0.94	0.85–1.05	0.282
PLR	0.99	0.99–1	0.022
TC	0.95	0.72–1.24	0.689
TG	1.01	1.01–1.02	<0.001
TyG	6.57	3.35–12.86	<0.001
HDL	0.29	0.66–1.37	0.040
AIP	33.51	7.1–158.23	<0.001
LDL	0.95	0.66–1.37	0.797
UA	1.00	1–1.01	0.143
Hcy	1.00	0.97–1.03	0.948
WBC	1.07	0.97–1.18	0.176
FBG	1.41	1.21–1.64	<0.001
Lenth	0.98	0.94–1.02	0.363
Diameter	0.96	0.79–1.17	0.684
Stent site	0.97	0.45–2.1	0.948
Hypertension	2.54	1.21–5.32	0.013
Diabetes mellitus	7.57	3.96–14.48	<0.001
CAD	1.69	0.7–4.08	0.240
Hyperlipidaemia	1.21	0.32–4.54	0.782
ADP	1.01	0.99–1.04	0.278
Cycle	1.38	0.75–2.52	0.296
Antiplatelet therapy	0.52	0.29–0.95	0.035
BMI	1.17	1.08–1.28	<0.001
Innihss	1.06	1–1.12	0.040
Outnihss	1.21	1.05–1.4	0.010
InMRS	1.33	1.06–1.66	0.012
OutMRS	1.45	1.06–2	0.021

NIHSS, National Institutes of Health stroke scale; MRS, modified Rankin scale; ISR, in-stent restenosis; TG, triglyceride; TC, total cholesterol; LDL, low-density lipoproteins; HDL, high-density lipoproteins; HCY, homocysteine; WBC, white blood cells; ADP, Adenosine Diphosphate; UA, uric acid; TyG, triglyceride-glucose index; AIP, atherogenic Index of Plasma, antiplatelet therapy ticagrelor or clopidogrel.

**Table 3:** Collinearity assessment results.

Variables	VIF
TG	8.722
TyG	12.465
FBG	5.614
Diabetes Mellitus	1.317

**Table 4:** Predictive factors for ISR.

Variables	Coefficient	Standard error	Wald	OR	95 %CI	*p* value
TyG	1.532	0.385	3.983	4.63	2.17–9.84	<0.001
DM	1.469	0.369	3.980	4.34	0.11–8.95	<0.001
Antiplatelet therapy	−0.810	0.376	−2.145	0.44	0.21–0.93	0.031
BMI	0.124	0.056	2.229	1.13	1.01–1.26	0.026
InMRS	0.276	0.136	2.030	1.32	1.01–1.72	0.042

TyG, triglyceride-glucose index; DM, Diabetes mellitus, antiplate-treatment ticagrelor or clopidogrel; MRS, modified Rankin scale.

### Construction and validation of prognostic nomogram

**Figure 2:**
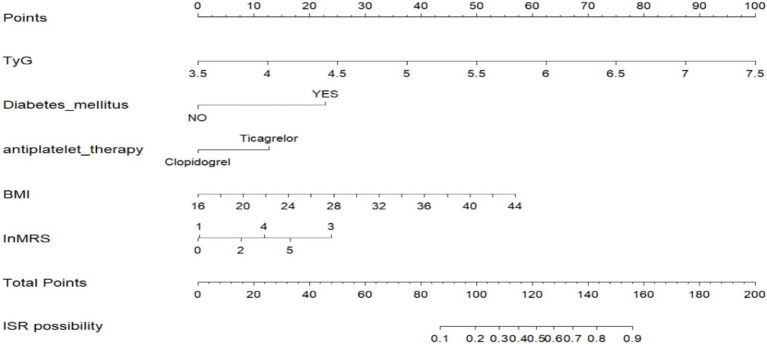
Establishment of a nomogram model for predicting in-stent restenosis in patients.

**Figure 3:**
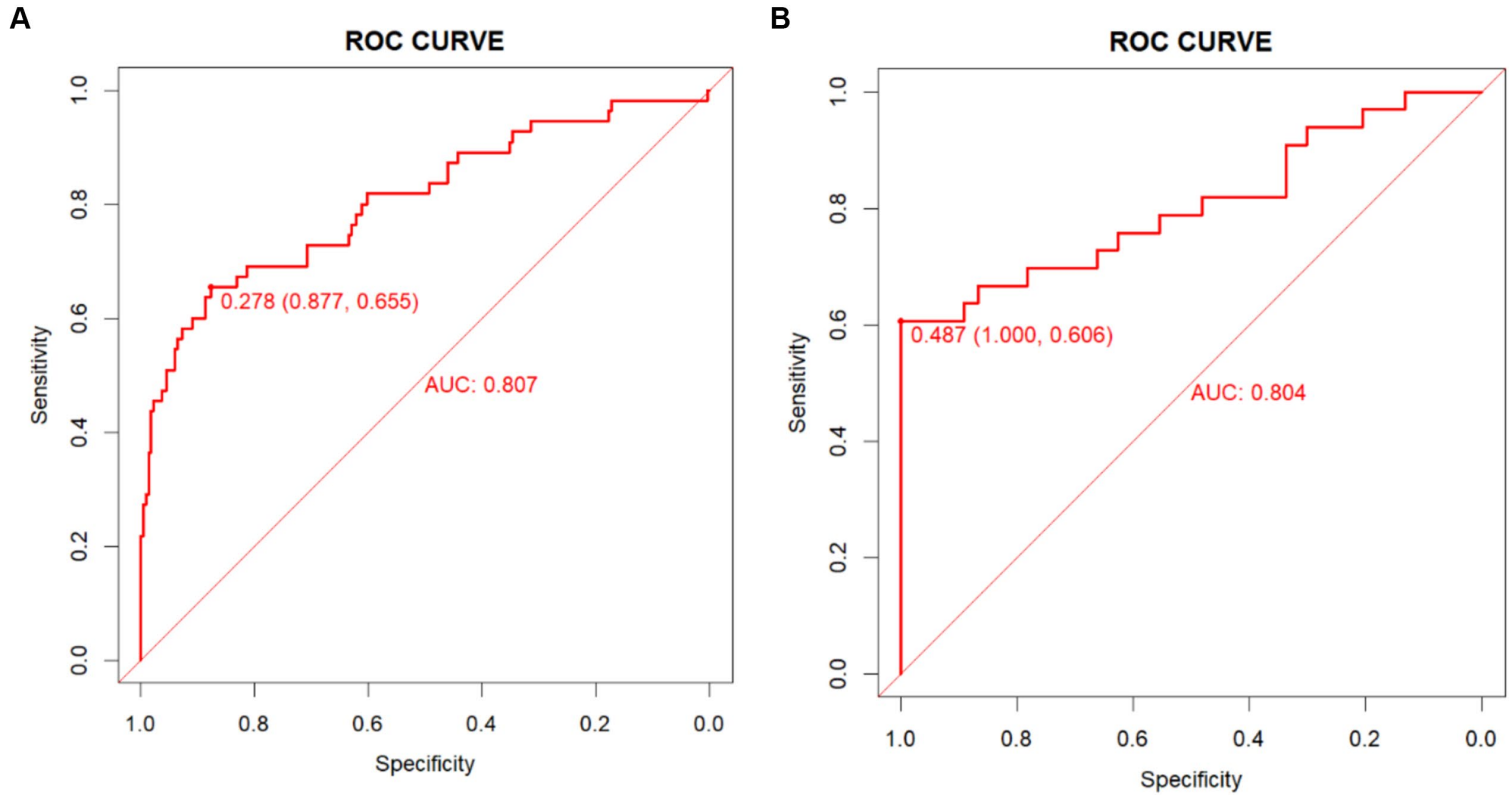
Evaluation of the discriminative ability of the nomogram. **(A)** The ROC curves and AUC values in the training in the training group. **(B)** The ROC curves and AUC values in the validation in the validation group.

**Figure 4:**
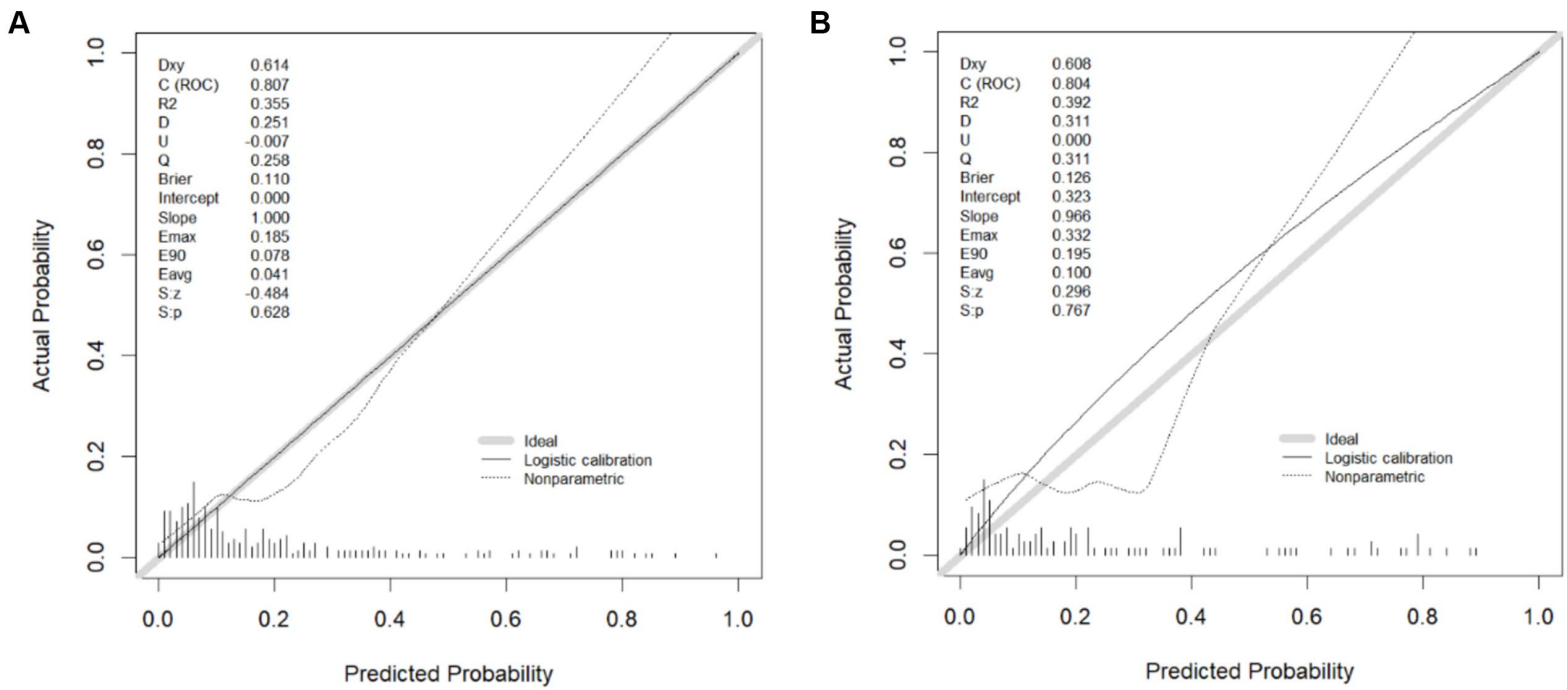
The calibration curve of in-stent restenosis for the training cohort **(A)** and the validation cohort **(B)**. The *x*-axis is the probability of incidence and the *y*-axis is actual incidence. Gray lines represent perfect calibration models, where predicted and actual probability are equal.

**Figure 5:**
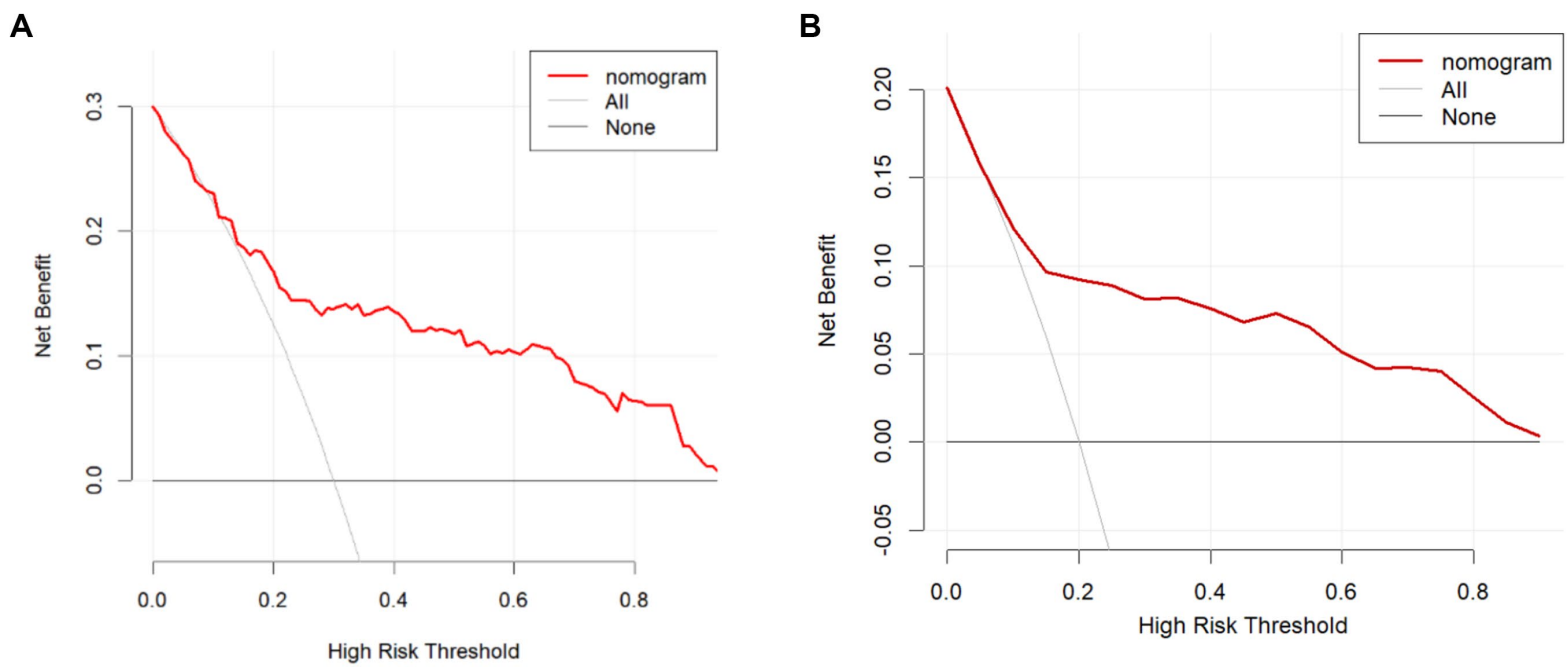
The DCA of the nomogram. **(A)** Decision Curve Analysis (DCA) for the training group. **(B)** Decision Curve Analysis (DCA) for the validation group.

A nomogram model was established based on risk factors derived from multivariable logistic regression analysis (Figure 2). The ROC AUC for predicting in-stent restenosis from the nomogram is 0.807, respectively, in the training group and 0.804, in the validation group (Figure 3). The C-index of the nomogram was 0.807 (95%CI = 0.655–0.877) in the training group and 0.804 (95% CI = 0.606–1.000) in the validation group. The calibration curve of the nomogram indicates that there is good consistency between the actual and predicted incidence of ISR in both the training group and validation group (Figure 4). In summary, the nomogram for ISR demonstrates strong discriminative and calibration abilities. DCA in both the training and validation groups shows that the nomogram has good clinical performance (Figure 5). DCA demonstrated a net clinical benefit of the In-Stent Restenosis-based nomogram across a broad range of threshold probabilities.

## Discussion

In this study, among the 390 patients who successfully underwent cerebral artery stenting, follow-up DSA at 6 months revealed an ISR incidence of 23.6%. This incidence is slightly higher than the 5–20% restenosis rate reported in the ICSS (International Carotid Stenting Study) trial (10). However, some reports have indicated that the incidence of ISR can range from 6 to 40.7% (23, 24). The occurrence of ISR can lead to more severe ischemic events, making accurate prediction of ISR a key approach to improving long-term patient outcomes. Although some models can predict stroke recurrence, allowing for effective preventive measures to be implemented for patients in advance, predicting ISR remains crucial for long-term management. For instance, the Framingham Stroke Profile (FPS) can predict the probability of stroke occurrence within 10 years, helping individuals determine whether interventions are needed (25). However, it is not a model designed to predict the occurrence of ISR. The ABCD score is primarily used to predict the risk of stroke recurrence within 90 days, providing information on the patient’s short-term prognosis (26). These models focus on predicting stroke recurrence risk and involve a wide range of complex factors. The SAMMPRIS-ISR standard provides an important evaluation tool for in-stent restenosis after intracranial stenting (20). Using a single, unified model may not ensure accurate predictions for all patients. However, our model specifically predicts the incidence of ISR, reducing interference from other factors, which can enhance the accuracy of the predictions. Additionally, the model focuses on patients who have successfully undergone stent implantation, a population with similar characteristics, demonstrating homogeneity. The predictive factors used in the model are common clinical indicators that are easily accessible, making the model practical for direct application in clinical settings. This study found that TyG, presence of Diabetes Mellitus, postoperative dual antiplatelet therapy, BMI, and preoperative MRS score are risk factors for the occurrence of ISR in patients after stent implantation. The C-index of our model was 0.807 (95%CI = 0.655–0.877) in the modeling group and 0.804 (95% CI = 0.606–1.000) in the validation group, This indicates that the model demonstrates good discriminatory ability. The calibration curve indicates that the predicted occurrence of ISR aligns well with the actual outcomes, demonstrating good consistency. DCA suggests that the nomogram offers a net clinical benefit, providing valuable reference for clinical practice. It can help physicians more accurately predict the risk of ISR in patients, identify those at risk early on, and tailor personalized treatment measures to reduce the incidence of ISR, ultimately improving patient outcomes.

Diabetes is associated with an increased prevalence and severity of carotid artery disease (27), it is a significant risk factor for ischemic stroke (28). Casana’s study found that the incidence of ISR in diabetic patients was significantly higher than in non-diabetic patients, reaching as high as 21.2% (29). The mechanism by which diabetes contributes to ISR may involve accelerating the formation of neointimal hyperplasia within the stent (12). Comparable mechanisms indicating accelerated restenosis in diabetic patients have also been identified in other areas of interventional research (30). Relevant studies have found that after stent implantation, atherosclerotic plaques do not rupture but are pushed outward to fmodified to the media, which subsequently stimulates neointimal hyperplasia (31). At the same time, stent implantation can also cause some damage to the endothelium. Elevated blood glucose levels result in an increase in non-enzymatic glycation end products and an elevation of oxygen and nitrogen free radicals within the mitochondrial respiratory chain, creating an imbalance between oxidants and antioxidants. In the presence of various inflammatory mediators, this impairs the repair of damaged cells. Additionally, it can lead to impaired coagulation function and thrombus deposition, ultimately resulting in lumen narrowing within the stent (32). It is clear that maintaining optimal blood glucose levels and actively managing blood sugar can effectively reduce the incidence of ISR.

The TyG index has been validated as an independent prognostic factor in cardiovascular diseases and can also predict the occurrence of in-stent restenosis in patients undergoing stent treatment for acute coronary syndrome (33, 34). A study from Tangdu Hospital of Air Force Military Medical University found that the TyG index also has predictive value for in-stent restenosis in cerebrovascular conditions, showing a positive correlation with the risk of restenosis (13). The TyG index is used to assess insulin resistance, and extensive evidence has demonstrated that insulin resistance is closely associated with the development of cerebrovascular diseases (13). The effects of insulin on blood vessels are quite complex. Physiologically, insulin activates the PI3K pathway, which encourages endothelial cells to generate nitric oxide (NO). This process inhibits smooth muscle cell migration, prevents neointimal hyperplasia, and decreases platelet adhesion and aggregation. However, in the context of ISR, insulin downregulates the PI3K/AKT pathway while primarily activating the MAPK pathway, leading to a decrease in NO release. This decrease promotes smooth muscle proliferation, cell migration, and plaque formation. Additionally, insulin resistance (IR) triggers the release of pro-inflammatory factors and free fatty acids, which may also contribute to ISR (35, 36). These studies provide indirect support for our results and underscore the necessity for broader clinical use of the TyG index in evaluating prognosis and tracking disease progression.

For patients undergoing intracranial stent placement, dual antiplatelet therapy is routinely administered, gradually transitioning to monotherapy with aspirin (37). Common dual antiplatelet regimens include aspirin-clopidogrel and aspirin-ticagrelor. Ticagrelor is an antiplatelet medication that differs from clopidogrel in that it binds reversibly to the P2Y12 receptor, thereby inhibiting platelet aggregation. Its efficacy is significantly superior to that of clopidogrel in certain cardiovascular interventional procedures, demonstrating higher effectiveness and stronger platelet inhibition (38, 39). The efficacy of clopidogrel varies among individuals, with approximately 58.8% of the Asian population not achieving the expected therapeutic effect. Some patients may even experience serious events such as stroke or myocardial infarction due to clopidogrel resistance (40, 41). The large-scale CHANCE-2 trial indicated that the combination of aspirin-ticagrelor significantly reduces the risk of stroke recurrence compared to aspirin-clopidogrel. However, the study did not address whether this combination has any impact on intracranial stents (42). This study found that patients on the clopidogrel regimen had a higher likelihood of developing ISR, which aligns with the observations made in the aforementioned research. Ticagrelor has been shown to have a safety profile comparable to that of clopidogrel. Therefore, in the absence of relevant contraindications and adverse effects, the combination of aspirin-ticagrelor is recommended as the preferred treatment regimen for patients after stent placement.

BMI reflects whether a patient’s weight is within a normal range and also indicates the level of body fat accumulation. A meta-analysis revealed that patients with BMI in the obese range have a significantly higher risk of developing ISR compared to those with normal or underweight BMI (43). Research has shown that obesity, aside from being a risk factor for conditions like hypertension and diabetes, is also linked to insulin resistance, pro-inflammatory states, and endothelial dysfunction (44, 45). Additionally, the associations between diabetes, inflammation, neointimal hyperplasia, and ISR have also been well-established. Therefore, it can be inferred that the mechanisms by which obesity leads to ISR may be multifaceted. The primary underlying mechanisms are that obesity leads to dyslipidemia, increasing blood viscosity and reducing blood flow velocity (46). Additionally, adipocytes secrete inflammatory factors, and inflammation plays a key role in the process of neointimal hyperplasia (47). ISR primarily results from endothelial damage and abrasion caused by balloon expansion and stent placement. This vascular injury triggers the release of mediators that promote the adhesion of platelets, neutrophils, and monocytes. These cells release substances that are vasoactive, thrombogenic, lymphocytic, and mitogenic, leading to vasoconstriction, vascular remodeling, neointimal growth, thrombosis, and inflammation, which ultimately contribute to ISR (7). Our study results indicate that as BMI increases, the incidence of ISR rises, which is generally consistent with previous research findings.

The MRS is primarily used to assess a patient’s neurological function status, the preoperative MRS score can evaluate the functional status of a patient’s nervous system at the time of stent placement. A higher MRS score indicates worse functional status in patients, reflecting the potential presence of significant issues, such as severe inflammatory responses. This may explain why preoperative MRS scores can predict the occurrence of ISR in patients. MRS scores are readily obtainable in clinical practice, as nearly all patients are evaluated by qualified healthcare professionals. For patients with higher scores, physicians can develop personalized treatment plans to reduce the incidence of ISR and effectively improve patient outcomes.

## Conclusion

This study established a predictive model that includes five variables: TyG, presence of Diabetes Mellitus, postoperative dual antiplatelet therapy, BMI, and preoperative MRS score. These variables can be used to predict the risk of ISR in patients following cerebral artery stenting. Internal validation demonstrated that the predictive model has good consistency. This model can better assist physicians in formulating personalized treatment plans for patients after stent implantation by addressing relevant risk factors and reducing the risk of ISR. To further validate the predictive performance of this model, more extensive external validation is needed, particularly through multicenter studies that include a larger and more diverse population.

## Limitations

Despite its contributions, this study has several limitations. First, it relies on data from a single-center cohort. Second, the clinical prediction model is based on a dataset with a limited sample size, highlighting the need for a larger population to enhance its credibility and reliability. Moreover, although internal validation showed strong agreement with the final model, findings should be interpreted with caution due to the lack of external validation, which is crucial for evaluating generalizability. Therefore, further research involving external validation across different populations and prospective cohort studies is necessary to substantiate these findings. Lastly, given the distinct mechanisms of intracranial versus extracranial in-stent restenosis, potential heterogeneity cannot be ruled out despite the lack of statistical significance in the our study.

## Data Availability

The original contributions presented in the study are included in the article/supplementary material, further inquiries can be directed to the corresponding author.
