# Correction: Establishment and validation of a clinical prediction model for in-stent restenosis after intracranial and extracranial stent implantation

**DOI:** 10.3389/fneur.2025.1686487

**Published:** 2025-09-03

**Authors:** Xiaohan Liang, Kuochang Yin, Yidian Fu, Guodong Xu, Xiaoxiao Feng, Peiyuan Lv

**Affiliations:** ^1^Graduate School of Hebei North University, Zhangjiakou, Hebei, China; ^2^Department of Neurology, Hebei General Hospital, Shijiazhuang, Hebei, China; ^3^Graduate School of Hebei Medical University, Shijiazhuang, Hebei, China; ^4^Hebei Provincial Key Laboratory of Cerebral Networks and Cognitive Disorders, Shijiazhuang, China; ^5^Department of Neurology, Shijiazhuang People's Hospital, Shijiazhuang, Hebei, China

**Keywords:** restenosis, nomogram, clinical prediction model, cerebral artery stent implantation, TyG

The affiliation “Department of Neurology, Hebei General Hospital, Shijiazhuang, Hebei, China” was omitted for author Xiaohan Liang. This affiliation has now been added.

The original version of this article has been updated.

